# Seed Priming with the Selenium Nanoparticles Maintains the Redox Status in the Water Stressed Tomato Plants by Modulating the Antioxidant Defense Enzymes

**DOI:** 10.3390/plants12071556

**Published:** 2023-04-04

**Authors:** Muhammad Ishtiaq, Muhammad Waqas Mazhar, Mehwish Maqbool, Tanveer Hussain, Syed Atiq Hussain, Ryan Casini, Ahmed M. Abd-ElGawad, Hosam O. Elansary

**Affiliations:** 1 Department of Botany, Mirpur University of Science and Technology, Mirpur 10250, Pakistan; 2 Department of Botany, University of Gujrat, Gujrat 50700, Pakistan; 3 School of Public Health, University of California, Berkeley, 2121 Berkeley Way, Berkeley, CA 94704, USA; 4 Department of Plant Production, College of Food & Agriculture Sciences, King Saud University, P.O. Box 2460, Riyadh 11451, Saudi Arabia

**Keywords:** selenium nanoparticles, tomato, drought stress, ascorbate glutathione, antioxidant defense

## Abstract

In the present research, selenium nanoparticles (SeNPs) were tested for their use as seed priming agents under field trials on tomatoes (*Solanum lycopersicum* L.) for their efficacy in conferring drought tolerance. Four different seed priming regimes of SeNPs were created, comprising 25, 50, 75, and 100 ppm, along with a control treatment of 0 ppm. Seeds were planted in split plots under two irrigation regimes comprising water and water stress. The results suggest that seed priming with SeNPs can improve tomato crop performance under drought stress. Plants grown with 75 ppm SeNPs-primed seeds had lower hydrogen peroxide (H_2_O_2_) and malondialdehyde (MDA) levels by 39.3% and 28.9%, respectively. Seed priming with 75 ppm SeNPs further increased the superoxide dismutase (SOD) and catalase (CAT) functions by 34.9 and 25.4%, respectively. The same treatment increased the total carotenoids content by 13.5%, α-tocopherols content by 22.8%, total flavonoids content by 25.2%, total anthocyanins content by 19.6%, ascorbic acid content by 26.4%, reduced glutathione (GSH) content by 14.8%, and oxidized glutathione (GSSG) content by 13.12%. Furthermore, seed priming with SeNPs upregulated the functions of enzymes of ascorbate glutathione cycle. Seed priming with SeNPs is a smart application to sustain tomato production in arid lands.

## 1. Introduction

The amount of water available for agricultural irrigation decreases as a result of reservoirs getting smaller due to rising temperatures, a trend that is getting stronger over time [1]. In a number of rain-fed agricultural regions around the world, the yearly cumulative precipitation has decreased as a result of global warming [2]. Around one-fifth of the world’s population will be adversely impacted by water scarcity in such a scenario, assuming the predicted increase in air temperature of roughly 2 °C above current levels by the end of the century [3].

Suboptimal water availability due to changing climates is affecting global crop production and increasing food security risks [4]. Limited water availability leads to oxidative damage to the plants due to the production of reactive oxygen species (ROS) in various cellular compartments such as mitochondria, peroxisomes, and chloroplasts [5]. The production of hydroxyl radicals, singlet oxygen, H_2_O_2_, and peroxide anion as a result of limited water supply is harmful to plants, reducing yield and production [4]. The excessive ROS leads to the departure of plants from normal homeostasis, including nucleic acid destruction, enzyme denaturation, lipid peroxidation, and carbohydrate deoxidation [6].

Plants have innate defense mechanisms to combat lower water availability, including concentrating compatible solutes, upregulating antioxidant defense enzymes, increasing non-enzymatic antioxidants, and mitigating stress through enzymatic pathways such as the ascorbate glutathione cycle and glutathione peroxidase cycle [7]. The maintenance and protection of the osmotic state by the plants activates ROS scavenging mechanisms and thereby helps to maintain redox status in the plants, leading to the induction of a stress tolerance response [8]. Plants facing water stress accumulate compatible solutes such as proline and enhance the quantities of secondary metabolites, which are key determinants of the physiological well-being of the plants exposed to abiotic stresses [9]. Under conditions of suboptimal water availability, there is an increase in the activities of the enzymes involved in ROS scavenging. For instance, abiotic stress leads to upregulation of enzymes involved in the ascorbate-glutathione cycle [10], increasing the detoxification of H_2_O_2_ produced as a result of increased photorespiration under conditions of limited supply. However, under prolonged droughts, there is a need to improve plants’ resistance to drought, as a limited supply of water may reduce the yield and production of plants by 70% [11].

Like all crop species, horticultural crops are facing decreased production due to the non-availability of water [12]. The tomato is the second most significant vegetable that is widely farmed and consumed worldwide in the solanaceous family, behind the potato [13]. Tomato production has a significant economic value in Pakistan [14,15]. In some areas of the country, tomatoes are produced throughout most of the year. However, supplies are significantly reduced during the summer’s high heat, from June to August. Based on the previous ten-year average, the current national tomato output is 10.1 t/ha, which is relatively low [15]. Similar to other crops, the tomato has a physiological, biochemical, and anatomical structure that is significantly inhibited by water constraints [16].

Tomatoes are extremely sensitive to water shortages, especially during the germination, seedling growth, and seed stage [16]. The literature reports that drought stress leads to poor growth and production of tomatoes due to decreased photosynthesis as a result of chlorophyll damage [17]. Drought stress leads to degradation of the envelop structure of chloroplasts due to the formation of H_2_O_2_, as evidenced by the enhanced accumulation of MDA [18]. To combat excessive ROS, tomato boosts its antioxidant defense system [19]. The antioxidant defense enzymes such as APX are involved in the detoxification of H_2_O_2_ through the ascorbate-glutathione pathway [20]. Furthermore, the tomato plants increase the accumulation of compatible solutes such as glutathione, flavonoids, and proline to maintain water potential. These osmolytes perform a key role in stress signaling and mitigation [21]. Therefore, there is a need for climate-smart and efficient techniques such as seed priming that may increase the growth and production of tomatoes. The pre-sowing hydration method called “seed priming” creates a physiological state that promotes uniform seedling emergence and triggers germination. Furthermore, seed priming has been linked to enhanced molecular, biochemical, and morpho-physiological responses in plants [22].

For a plant to operate normally, selenium (Se) is a crucial trace element. Fascinatingly, Se has been shown to be useful in reducing the negative effects on plants of environmental challenges such as heavy metals, salt, and infections. When administered in small amounts, Se works as an antioxidant and encourages plant development, but when applied in large doses, it may inhibit growth. An appropriate Se concentration can aid plants in maintaining membrane fluidity and structure and can also shield cellular organs from harm [23].

Due to their small size and distinctive surface properties, nanoparticles present an appealing approach for regulating agricultural production systems. By maximizing the nutrient supply and boosting the antioxidant defense system, slow nutrient release from nanoparticles may encourage plant development. SeNPs, which can enhance plant performance in challenged situations by boosting their growth recovery, are one type of nanoparticle that has recently attracted more attention [24]. As the research is progressing, numerous evidence are being reported for the role of SeNPs in boosting antioxidant defense in plants. Garza-Garca et al. [25] presented a review on SeNPs and documented that SeNPs upregulate the activity of antioxidant defense enzymes in plants such as APX, POD, CAT, and SOD. Application of SeNPs increases the activities of the phenylalanine ammonium lyase (PAL) enzyme, which is involved in the synthesis of secondary metabolites in plants through the phenylpropanoid biosynthetic pathway [26]. The literature reports the use of SeNPs in boosting antioxidant defense enzymes in citrus [27]. Furthermore, Shahbaz et al. [28] have reported the use of SeNPs in the upregulation of the antioxidant defense response in Mangifera indica. Additionally, the application of SeNPs for alleviation of heat stress in Sorghum bicolor has been reported [29].

Although exploration of SeNPs application has been reported in terms of its use in controlling late blight of tomato [30] and saline stress [31], the use of SeNPs as seed priming of tomato has not been documented. The exact mechanism by which SeNPs control the physiological, morphological, and biochemical alterations in tomato under water deficit circumstances is still unknown. As a result, the purpose of this study is to examine the effects of Se NPs in improving drought tolerance as well as to examine the mechanism by which SeNPs increased drought tolerance. Our primary goal was to look into the effects of drought stress on plant growth, tomato seedling membrane damage, osmolyte build-up, and the ROS homeostasis controlled by SeNPs. We specifically looked at enzymes connected to the Halliwell Asada pathway. The goal of this study is to further our understanding of the biological role of SeNPs in enhancing resistance to drought stress. Our hope is that tomato plants will have higher antioxidant defenses upon seed priming with Se nanoparticles. As a result, we hypothesize that seed priming with SeNPs may help tomato plants fight off drought stress by enhancing their antioxidant defense and enhancing accumulation of osmolytes. With tomato plants grown from primed seeds, we anticipate improved growth and output in yield form. The study’s goal is to look into the role of SeNPs as a potential seed pro-nutrient and its ability to prime seeds for better crop health and yield.

## 2. Results

**Figure 1:**
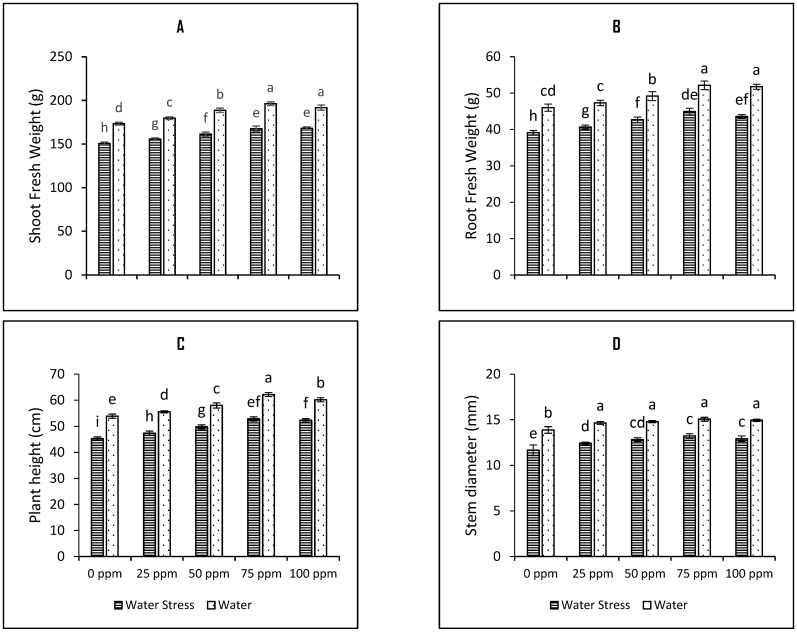
Bar charts (mean ± S.E; *n* = 3) representing growth attributes (**A**) shoot fresh weight (**B**) root fresh weight (**C**) plant height and (**D**) stem diameter of tomato plants raised from SeNPs-primed seeds grown under water stress and water conditions. 0, 25, 50, 75, and 100 ppm on *x*-axis represent concentrations of Se-NPs followed in the seed priming experiment. Bars with different alphabets represents mean data which significantly differs at (*p* < 0.05).

Drought stress reduced growth and biomass of the tomato plants; however, seed priming with SeNPs significantly increased growth and biomass of the experimental plants under both irrigation regimes (i.e., water and water stress). The shoot and root fresh weights were reduced significantly upon the imposition of drought stress by 13.09% and 14.7%, respectively, compared to control (Figure 1A,B). Seed priming with 75 ppm SeNPs increased the shoot and root fresh weights of water-stressed tomato plants by 11.2% and 13.9%, respectively. Furthermore, the growth of the plants in terms of plant height and stem diameter was improved by seed preconditioning with SeNPs. Drought stress reduced plant height and stem diameters of the experimental plants by 15.9% and 16.06%, respectively. Seed priming with 75 ppm SeNPs improved the plant height by 16.6% and the stem diameter of tomato plants by 13.4% and was the best among all tested seed priming regimes (Figure 1C,D). The treatments were found to be significant as tested by the results of the least significant difference (LSD) scores. The two-way analysis of variance (ANOVA) provided the significance of priming treatments at a *p*-value below 0.001 (Figure 1; Table 1).

**Figure 2:**
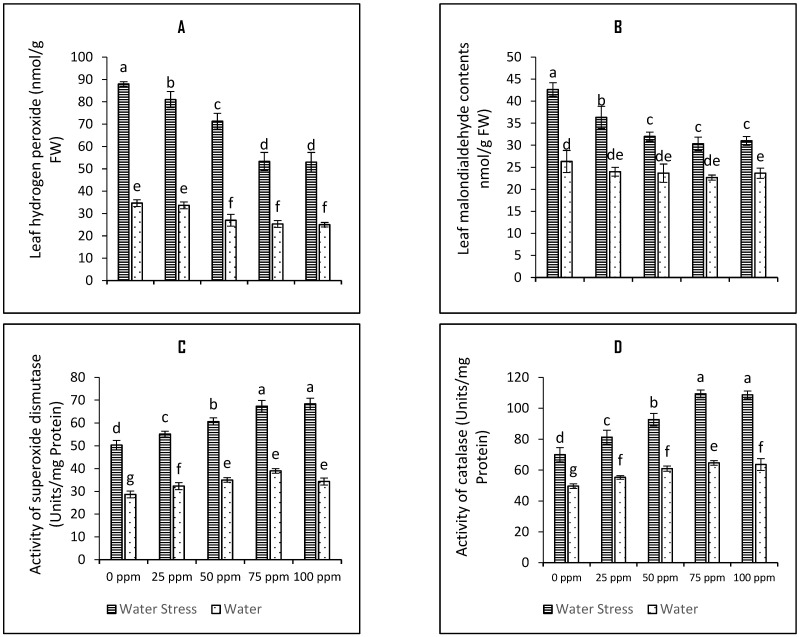
Bar charts (mean ± S.E; *n* = 3) representing levels of osmotic stress markers (**A**) leaf hydrogen peroxide contents and (**B**) leaf malondialdehyde contents and functioning of antioxidant enzymes (**C**) superoxide dismutase activity and (**D**) catalase activity in tomato plants raised from SeNPs-primed seeds grown under water stress and water conditions. 0, 25, 50, 75, and 100 ppm on *x*-axis represent concentrations of Se-NPs followed in the seed-priming experiment. Bars with different alphabets represents mean data which significantly differs at (*p* < 0.05).

To predict the stress level on tomato plants, the contents of H_2_O_2_ (a form of ROS) were evaluated (Figure 2A). The biological membranes damage due to the formation of ROS as result of lipid peroxidation due to abiotic stress were measured on the basis of MDA accumulation (Figure 2B). Drought stress increased the level of MDA by 62%. Seed priming with SeNPs mitigated the levels of these stress markers and all seed priming regimes were found beneficial; however, the 75-ppm concentration of SeNPs was best among all treatments. The plants raised through 75 ppm SeNPs-primed seeds experienced reduced H_2_O_2_ and MDA contents by 39.3% and 28.9%. The interaction in case of both variables was found significant (*p* < 0.001) as mentioned in Table 1.

Activities of the antioxidative defense enzymes SOD and CAT increased significantly by 40.9% and 75.5% respectively under drought stress. All the tested priming regimes were found significant in increasing the functioning of SOD and CAT. Seed priming with 75 ppm SeNPs further increased the SOD and CAT functioning by 35.9% and 25.4% respectively (Figure 2C,D). ANOVA predicted significant interaction at *p*-value below 0.001.

**Figure 3:**
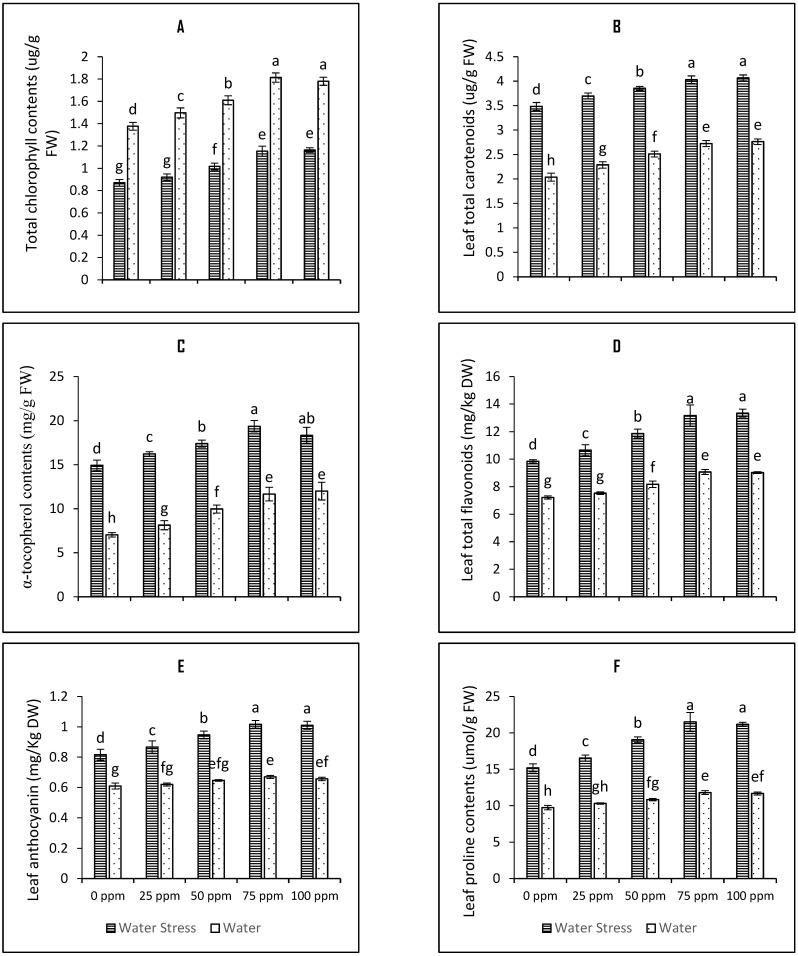
Bar charts (mean ± S.E; *n* = 3) representing photosynthetic pigments, and assayed antioxidant metabolites (**A**) total chlorophyll contents (**B**) leaf total carotenoids (**C**) leaf tocopherol contents (**D**) leaf total flavonoids (**E**) leaf anthocyanin and (**F**) leaf proline contents in tomato plants raised from SeNPs-primed seeds grown under water stress and water conditions. 0, 25, 50, 75, and 100 ppm on *x*-axis represent concentrations of Se-NPs followed in the seed-priming experiment. Bars with different alphabets represent mean data which significantly differs at (*p* < 0.05).

The contents of total chlorophyll were reduced by 36.8% in the tomato leaves upon imposition of drought stress. Seed priming with SeNPs enhanced total chlorophyll contents and all the treatments proved beneficial under both irrigation regimes; however, the 75 ppm concentration of SeNPs was the best among all in raising total chlorophyll values (Figure 3A). The said treatment increased the total chlorophyll contents by 32.5% compared to respective control under drought stress. The ANOVA predicted a significant *p* value at alpha 0.05% (Table 1).

**Figure 4:**
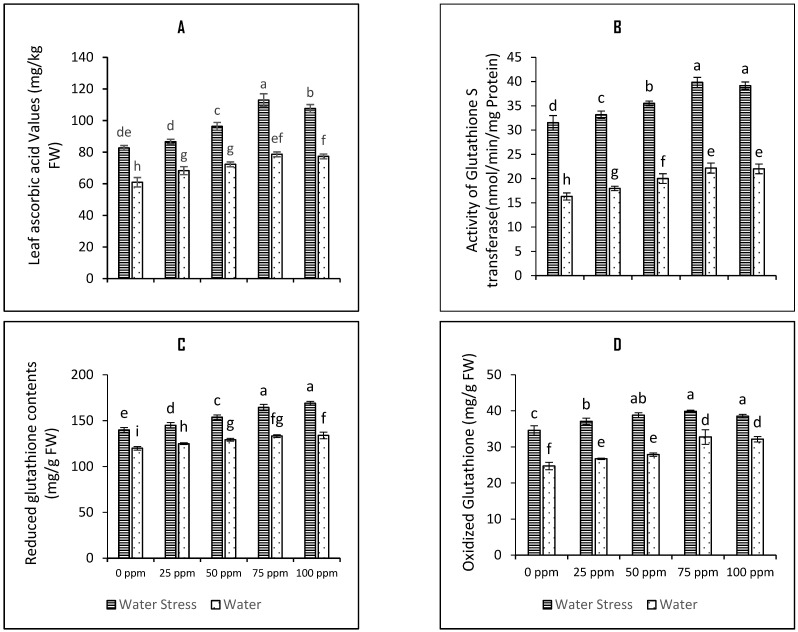
Bar charts (mean ± S.E; *n* = 3) representing activity of glutathione s transferase, and contents of ascorbic acid and glutathione in tomato plants raised from SeNPs-primed seeds grown under water stress and water conditions where: (**A**) leaf ascorbic acid contents (**B**) glutathione s transferase activity (**C**) reduced glutathione contents and (**D**) oxidized glutathione. 0, 25, 50, 75, and 100 ppm on *x*-axis represent concentrations of Se-NPs followed in the seed priming experiment. Bars with different alphabets represents mean data which significantly differs at (*p* < 0.05).

**Figure 5:**
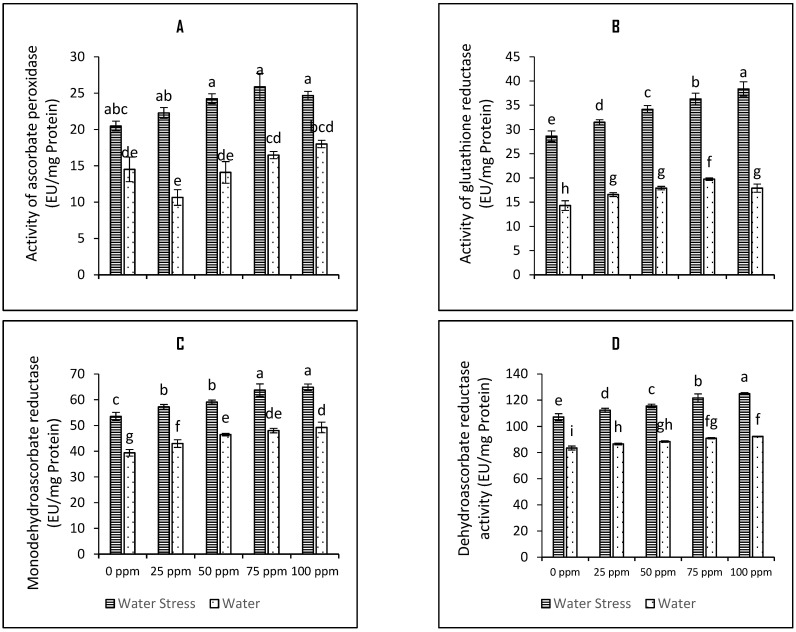
Bar charts (mean ± S.E; *n* = 3) representing functioning of four enzymes involved in ascorbate-glutathione cycle (**A**) ascorbate peroxidase (**B**) glutathione reductase (**C**) monodehydroascorbate reductase and (**D**) dehydroascorbate reductase for tolerance against water stress in tomato plants raised from SeNPs-primed seeds. 0, 25, 50, 75, and 100 ppm on *x*-axis represent concentrations of Se-NPs followed in the seed priming experiment. Bars with different alphabets represents mean data which significantly differs at (*p* < 0.05).

The activities of non-enzymatic antioxidants such as total carotenoids, α-tocopherols, total flavonoids, total anthocyanins, ascorbic acid, and glutathione (both oxidized and reduced forms of glutathione) were studied to explain their role in stress tolerance response by tomato plants. Our results suggest that imposition of drought stress increase the contents of total carotenoids, α-tocopherols, total flavonoids, total anthocyanins, ascorbic acid, GSH and GSSG by 71% (Figure 3B), 82% (Figure 3C), 36.3% (Figure 3D), 33.8% (Figure 3E), 35.5% (Figure 4A), 16% (Figure 4C), and 40% (Figure 4D) respectively. All the tested SeNPs seed priming concentrations were found beneficial in increasing the contents of these assayed non enzymatic antioxidants under both irrigation regimes. Seed priming with 75 ppm SeNPs further increased the contents of total carotenoids by 13.5%, the contents of α-tocopherols by 22.8%, the contents of total flavonoids by 25.2%, the contents of total anthocyanins by 19.6%, the contents of ascorbic acid by 26.4%, the contents of GSH 14.8%, and the contents of GSSG by 13.12% was observed. The mean square and *p*-values of all of these variables have been tabulated in the Table 1 of this manuscript, whereas the LSD score at alpha 5% have been presented in the bar charts for the variables as shown in the Figure 1, Figure 2, Figure 3, Figure 4 and Figure 5. The data presented in the Figure 4B reflects the increased activity of glutathione s transferase upon induction of drought stress on the tomato plants which further enhanced upon seed priming with SeNPs by 20.9%.

Leaf proline contents were evaluated and found significantly higher in the tomato plants exposed to drought stress. Seed priming further enhanced the contents of proline under both irrigation regimes. All the treatments were found effective (*p*-value < 0.001) as shown in Table 1. However, seed priming with 75 ppm SeNPs proved best among all seed priming regimes increasing the proline contents by 29% in the tomato plants grown under the water stressed environment (Figure 3F).

The functioning of ascorbate-glutathione cycle enzymes was assayed in the leaf of tomato plants followed in the current experiment. The results have been presented in Figure 5. The data presented in the Figure 5 suggests that activities of ascorbate-glutathione cycle enzymes are increased upon induction of drought stress. Drought stress increased the activity of APX by 41%, the activity of MDHAR by 35% and that of DHAR by 28%. The functioning of GR enzyme increased by two-fold upon induction of drought stress compared to respective control (0 ppm) of the irrigated plot plants. Seed priming with Se nanoparticles further boosted the activities of these enzymes. The water-stressed tomato plants raised through 75 ppm SeNPs exhibited further increase in the functioning of APX, GR, MDHAR, and DHAR by 20.8%, 21.1%, 16%, and 11.98% respectively (Figure 5; Table 1).

**Figure 6:**
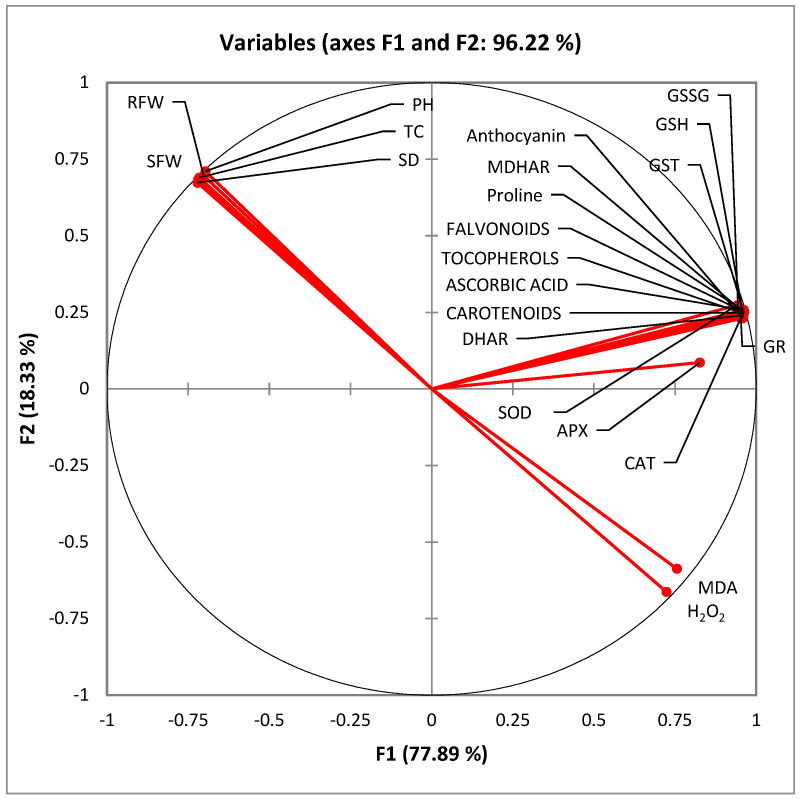
Principal component analysis (PCA) of various observed parameters followed in the study. RFW: root fresh weights; SFW: shoot fresh weights; PH: plant height; TC: total chlorophyll; SD: stem diameter; MDHAR: monodehydroascorbate reductase; DHAR: dehydroascorbate reductase; GST: glutathione s transferase; APX: ascorbate peroxidase; DR: glutathione reductase; CAT: catalase; MDA: malondialdehyde; H_2_O_2_: hydrogen peroxide; SOD: superoxide dismutase; GSH: reduced glutathione; GSSG: oxidized glutathione.

Principal component analysis (PCA) wagon wheel has been shown in Figure 6. There is aggregation of enzymatic, non-enzymatic antioxidants and enzymes of Asada-Halliwell pathway in the top right quarter of the wheel showing that drought stress increases these antioxidants. It is noteworthy that the contents and functioning of these antioxidants is further enhanced upon seed priming strengthening the antioxidants defense mechanism maintaining the redox status in tomato plants to cope with drought stress. In the top left quarter, there is aggregation of variables which are decreased upon induction of drought stress. These variables are improved upon seed priming. The lower quarter of loading chart shows the stress markers H_2_O_2_ and MDA which are increased upon imposition of drought stress and decreased upon seed priming with SeNPs. Spearman correlation matrix was constructed to interpret the correlations among the variables. The data presented in Table 2 demonstrates that all the variables are significantly correlated with each other either positively or negatively. The results of the study suggest that seed priming with the SeNPs maintains the redox status in the water stressed tomato plants by modulating the antioxidant defense enzymes and enzymes of ascorbate glutathione pathway.

## 3. Discussion

For reducing abiotic stresses and enhancing plant performance in these situations, there has recently been a rise in interest in employing nanotechnology in agriculture [22]. NPs’ effects on augmenting stress are greatly influenced by the plant under study, the doses of NPs used, the administration technique, and the type of stress. Therefore, in the present research, we tested various treatment concentrations of SeNPs and applied nanoparticles in the form of seed treatment. The results demonstrated an encouraging prospect in terms of stress resilience in tomato plants, and on most occasions, the 75 ppm concentration was found to be the best among all treatments. These results coincide with the previous work reported [22]. Similarly, seed priming with SeNPs improved tomato crop performance under well-irrigated conditions. Seed priming with SeNPs appears to be a biorational practice for improving tomato performance in both well-irrigated and water-stressed conditions.

Several studies have demonstrated that nanomaterials may be harmful when present in higher concentrations [32]. The best treatment for promoting aridity tolerance in tomato plants was found to be at 75 ppm on the majority of instances, and a 100 ppm concentration was found to be relatively less effective than 75 ppm on most occasions. Nanoparticles become poisonous at larger concentrations, and SeNPs toxicity is influenced by its concentration and solubility [33]. Higher concentration of Se might induce selenosis [34]. It may be assumed that might be the reason behind lower performance of tomato plants to 100 ppm concentration compared to 75 ppm treatment regime.

### 3.1. SeNPs-Mediated Seed Priming Increases Biomass and Photosynthetic Ability

The plant biomass in terms of plant height, stem diameter, and root and shoot fresh weights decreased upon drought imposition. Furthermore, there was a reduction in chlorophyll content in tomato leaves upon drought treatment [35]. Under the influence of drought, the excited pigments in the thylakoid membrane react, releasing hydroxyl radicals and H_2_O_2_ as forms of ROS. These substances result in the envelop peroxidation of chloroplasts, which weakens photosynthetic pigments and is connected to the inhibitory activity of chlorophyll enzymes such as chlorophyllase [36]. It might be assumed that drought stress imposition led to increased production of ROS, which resulted in decreased chlorophyll contents and ultimately lower biomass. Seed priming with SeNPs significantly increased tomato plant biomass and chlorophyll content. SeNPs promote the manufacture of photosynthetic pigments by safeguarding chloroplast enzymes and defending the chloroplast structure from severe oxidative damage, such as the destruction of grana and stromal lamellae [37]. Studies have shown that Se facilitates respiration and electron transport in the respiratory chain, speeding up the production of chlorophyll [38]. It can be assumed that increased biomass upon seed priming might be due to SeNPs mediating better photosynthesis in tomato plants under both tested irrigation regimes. These results were according to our expectations and proved our hypothesis that, under arid and semi-arid conditions, seed priming with SeNPs might be beneficial in increasing the growth and production of tomatoes. These results are in accordance with the previous results reported on the use of SeNPs in rice [39]. Additionally, Se encourages roots to be more delicate and branching and aids in increasing water and nutrient absorption [40], as shown by our results. Recent investigations indicate that Se interacts with certain nutrients, such as Cu, Mo, Zn, and I. It appears that these elements might have affected Se’s bioavailability to the experimental tomato plants and thus better nutrient homeostasis might have enabled tomato plants to perform better under both drought and water. Se and these nutrients generally follow a similar pattern with regard to their interactions with absorption and its transporters and the biological roles they carry out in plants [41]. This improved nutrient acquisition pattern might have enabled the tomato plants to confer drought tolerance upon seed priming with SeNPs. It might be assumed that SeNPs mediated increased root proliferation and associated nutrient acquisition enabled the tomato plants to survive better under lower water availability. These results are in accordance with the previous studies [42,43].

### 3.2. Impact on Antioxidant Defence Enzymes and Osmotic Stress Markers

Drought stress resulted in increased H_2_O_2_ and MDA contents [22]. Although plants require ROS production due to its role in cell proliferation, better seed germination, and pollen tube development, a delicate balance between ROS production and its detoxification is maintained within plants. When water is scarce, this balance is upset, and ROS production outpaces detoxification. It appears that drought-mediated overproduction of ROS led to increased contents of H_2_O_2_. The increased concentration of H_2_O_2_ led to enhanced MDA production. While maintaining a redox status, seed priming with SeNPs significantly reduced ROS content and lipid peroxidation in tomato plants. These findings were consistent with our expectations, and they show that seed priming with biorational nano formulations can be used to combat adverse conditions in fragile lands [44]. These results are in accordance with the previous work reported [22]. While decreasing the number of oxidative indicators in the examined tomato plants, SeNPs markedly boosted the activity of the oxidative enzymes SOD and CAT. Under stressful circumstances, SeNPs can function as antioxidants by triggering antioxidant defense mechanisms [45]. MDA levels are decreased in stressed plants by increasing the formation of antioxidant metabolites using Se products. Additionally, SeNPs change the lipid makeup of the membranes, which modifies their fluidity. By restoring the water potential lost due to drought and the relative water content of the leaves, priming affects osmotic adjustment and the potential of endo-membranous tissues [46]. It might be assumed that SeNPs mediated better water homeostasis and decreased the contents of ROS and MDA. A better antioxidant defense mechanism, on the other hand, leads to lower ROS levels, as demonstrated by our findings. CAT scavenges H_2_O_2_ by converting it into water, and SOD protects cells from abiotic stress by transforming superoxide radicals (a kind of ROS) into molecular oxygen. This priming mediated crosstalk between osmotic stress markers and antioxidative enzymes, enabling the tomato plants to survive better under water stress. It appears that seed priming enabled better ROS homeostasis in the tomato plants which enabled them to perform better under well-irrigated conditions. The antioxidant enzymes’ activity decreases with poor nutrient availability. Thus, it can be inferred that Se nanoparticle seed priming may have induced a stress tolerance response in tomato plants based on the improved activities of these antioxidant enzymes. This was quite apparent, as the findings of the present study reported decreased contents of H_2_O_2_ and a by-product of lipid peroxidation [47]. The SeNPs-mediated increased activities of antioxidant defense enzymes has been reported by several researchers [48,49,50].

### 3.3. Modulation of Ascorbate Glutathione Cycle Enzymes

In plants, glutathione-ascorbate cycle is involved in enhancing the glutathione pool and detoxification of H_2_O_2_. Reduced glutathione is created in the glutathione-ascorbate cycle from oxidized glutathione using given electrons from all antioxidants. Since drought stress leads to increased H_2_O_2_, plants activate their innate defense mechanism to mitigate this rise in ROS burst. This also leads to enhanced activities of ascorbate glutathione enzymes [51]. Tomato plants raised with SeNPs-primed seeds had a better antioxidant defense response, as well as increased APX activity. Rios et al. [52] reported similar results with Se application to lettuce plants.

Seed priming with SeNPs boosted the activity of GR, a flavoprotein which uses NADPH as an electron donor to conduct the reduction of glutathione disulfide. Flavin-adenine dinucleotide (FAD), an electron transporter with properties similar to NAD, is used as the electron acceptor by a class of oxidizing enzymes known as flavoproteins [53]. This molecule is noteworthy because it contains the vitamin riboflavin as a fundamental component. For the utilization and recycling of glutathione, Se and vitamin B2 (riboflavin) are crucial [54]. It might be assumed that SeNPs-mediated seed priming might have increased Se availability, which ultimately increased the activities of GR. An increased activity of MDHAR was observed in tomato plants raised through SeNPs-primed seeds. In plants, MDHAR reduces monodehydroascorbate to produce ascorbate. The increased concentration of ascorbic acid observed in the experiment might be due to increased MDHAR functioning. MDHAR belongs to a class of enzymes named oxidoreductases. When consumed physiologically, Se performs its role as a component of selenoproteins, the majority of which are oxidoreductases. Thus, it can be assumed that Se application might have resulted in increased activity of selenoproteins, which ultimately affected the functioning of MDHAR [55]. A marked increase in the activity of DHAR was observed upon Se priming. The reduced ascorbic acid or ascorbate is changed to dehydroascorbate, the oxidized form, during an enzymatic reaction or antioxidant activity, which is subsequently recycled back to ascorbate by DHAR [56]. Ascorbate works as an antioxidant in the body and as a cofactor in hydroxylation processes, keeping vitamin E (tocopherols) in its reduced, active state. As a result, the increase in tocopherols observed in our study could be attributed to increased DHAR activity [57]. Sun et al. [58] previously demonstrated that Se increases the activities of ascorbate-glutathione cycle enzymes. The nanoparticles-mediated increased activities of these enzymes have been documented by the previous researchers [59,60]. Under well-irrigated conditions, SeNPs also upregulated the activities of these enzymes. Apart from their role in stress mitigation, the antioxidants are crucial in membrane protection, proper seedling emergence, cell proliferation, and programmed cell death. It appears that these antioxidants might have enabled a proper homeostatic response in the tomato plants, enabling them to perform even better under well irrigated conditions.

### 3.4. SeNPs-Mediated Seed Priming Lifts Ascorbate Glutathione Substrate Pool

Drought stress led to more accumulation of ascorbic acid in the tomato plants. The negatively charged ion of the ascorbic acid is called ascorbate. Increased accumulation of ascorbic acid under drought has been reported by previous studies [61,62]. Similarly, there was an increase in glutathione pool upon drought stress imposition in the experimental tomato plants. The increase in ascorbic acid and glutathione is a natural defense response of plants under abiotic stress. A marked increase in ascorbic acid and glutathione was seen in tomato plants raised through SeNPs-primed seeds. These results are in accordance with a previous study reported by Rios et al. [52]. Many biological functions, including the synthesis of proteins and DNA, cell division and development, the metabolism of xenobiotics, the transport of amino acids, and redox-sensitive signal transduction, depend on cellular GSH. Thus, it might be assumed that enhanced glutathione levels under well irrigated as well as water stressed conditions is crucial to proper biological functioning within a plant. A number of ROS, such as hydroxyl radicals, hydroperoxides, alkoxyl radicals, and superoxide anions, can directly react with the thiolic group in GSH. As there are no enzymatic defenses against the hydroxyl radical, GSH functions as a free radical scavenger. It is particularly effective against this radical species [63]. With the addition of GSH, glutathione S-transferases (GSTs) quench reactive chemicals and shield the cell from oxidative damage. In the present study, an increased activity of GST was observed upon seed priming with SeNPs. It can be assumed that GST-mediated increased activities might have enabled the tomato plants in conferring drought tolerance. These results are in accordance with the previous reports [64,65].

### 3.5. SeNPs-Mediated Seed Priming Increases Nonenzymatic Antioxidants and Secondary Metabolites Conferring Drought Tolerance

Secondary metabolite production is a defensive mechanism system used by plants to increase their adoption in adverse situations. Drought stress resulted in increased contents of assayed secondary metabolites and non-enzymatic antioxidants such as glutathione, ascorbic acid, carotenoids, flavonoids, and tocopherols. The increased contents of these non-enzymatic antioxidants and metabolites are part of the internal defense mechanisms of tomato plants exposed to drought stress [66]. Seed priming with SeNPs conferred a further increase in the contents of these metabolites both under well-irrigated and water-deficit conditions. Furthermore, the concentration of proline was enhanced as a compatible solute, leading to a stress alleviation response in the tomato plants raised from the primed seeds. These results were according to our hypothesis and fulfilled our expectations. Furthermore, these results are supported by previous studies [67,68]. Under drought stress, increased concentrations of compatible solutes such as proline result in improved solute composition within the protoplasm [69]. It can be assumed that tomato plants have better survived due to enhanced proline contents. Additionally, an increase in fructose 1,6-bisphosphatase activity, a crucial enzyme in glucose metabolism, is associated with an increase in total sugars following the administration of Se [70]. As a result, increased secondary metabolite content can be attributed to SeNPs-mediated improved sugar metabolism. There was an increase in the contents of phenolics such as tocopherols, carotenoids, and flavonoids upon seed priming. Phenolic compounds have antioxidant properties in that they collect and reduce ROS, which prevents the oxidation of essential biomolecules and inhibits oxidative stress or lessens its effects on plant cells [71]. Applying stress-modulating NPs, such as SeNPs, appears to have lessened the negative impacts of water stress and membrane lipid peroxidation, which in turn affected the amount of phenolics in plant cells, thereby triggering a stress-mitigating response. According to studies, the use of SeNPs increased the rate of photosynthesis by reducing the negative effects of drought stress and activating light-harvesting complex II-related genes. It can be assumed that a better photosynthetic apparatus led to a better secondary metabolism, which boosted tomato defense under drought stress [72].

Furthermore, nanoparticle-mediated seed priming promotes Ca^+2^ signaling in plants. Ca^+2^ functions as a secondary messenger in plants and, as a result, alters transcriptional reprogramming, enhancing secondary metabolism [73]. More flavonoids, vitamins, glycosylates, and phenols are produced as a result of improved metabolism. Additionally, seed priming enhances the metabolism of starch, leading to a build-up of soluble sugars in tomato plants that function as osmolytes and increase drought tolerance. The levels of ROS are optimized by seed nano-priming with SeNPs. The secondary metabolism in plants is enhanced by ideal ROS concentrations. Optimizing ROS levels encourages the activation of other signaling molecules, such as jasmone acid, which has an impact on plants’ secondary metabolism [74]. According to our findings, this causes a greater concentration of plant secondary metabolites like flavonoids. In the current study, we observed a rise in anthocyanin content during drought stress, which was further augmented after SeNPs seed priming. Anthocyanins in plants are ROS scavengers that are activated by drought stress. Anthocyanin helps plants withstand drought stress by preventing the accumulation of ROS, which results in effective water homeostasis [75].

In the present research, we tested several concentrations of SeNPs as a seed priming agent on a high-commercial-value horticultural crop tomato, particularly when grown under drought stress. The study filled a gap in the literature by reporting on the use of SeNPs on horticultural crops. Although the role of SeNPs in rice has been documented [39], the study reported mainly germination and growth traits of rice, with little emphasis given on the efficacy of SeNPs in antioxidant defense. In the same manner, Sardar et al. [76] reported the use of SeNPs on Coriandrum sativum and documented the role of SeNPs in improving heavy metal stress tolerance, but the study mainly focused on mineral homeostasis and three testing concentrations of SeNPs were used. Additionally, the role of Se-NPs in regulating the enzyme activity of the ascorbate-glutathione pathway was little explored in the previous studies. Indeed, in the past few studies, the use of SeNPs has been reported in the mitigation of late blight disease of tomatoes [30] and saline stress [31]; however, the present research mainly focuses on drought stress. So far, our results have proved encouraging and fulfilled our expectations in bridging research gaps on the use of SeNPs in agriculture.

In a nutshell, seed priming with SeNPs proved beneficial in the mitigation of drought stress in tomatoes. The tomato plants raised from primed seeds promised better plant height, stem diameter, and biomass. This increase in growth and biomass is due to increased biosynthesis of chlorophyll and carotenoids. The MDA and H_2_O_2_ contents decreased after priming treatments, indicating an increase in antioxidant defense enzyme activity. Seed priming by SeNPs increased the pool of non-enzymatic antioxidants and resulted in the accumulation of more compatible solutes such as proline. The antioxidant vitamin contents, such as tocopherols and ascorbic acid, were enhanced, which enabled the tomato plants to better survive under the dry stress. Apart from the role of SeNPs, priming itself is a biorational and classic agricultural practice to fight off environmental stresses. Agriculture was revolutionized by the evolution of priming techniques over time. Agriculture has seen a great deal of use of halopriming, biopriming, hydropriming, and hormonal priming. But with the emergence of nano priming, agriculture can be more sustainable under the influence of climate change.

## 4. Materials and Methods

### 4.1. Experimental Setup and Treatment Plan

The investigation was carried out in a lab and a research area at the Government Graduate College Sarai Alamgir in Gujarat, Punjab, Pakistan. The experiment was conducted in the field in accordance with [22]. Roma tomato seed was purchased from a nearby market for agricultural supplies. This cultivar is predicted to yield 79 g of fruit. After being surface sterilized for two minutes with 70% ethanol, the seeds were rinsed five times with distilled water.

**Figure 7:**
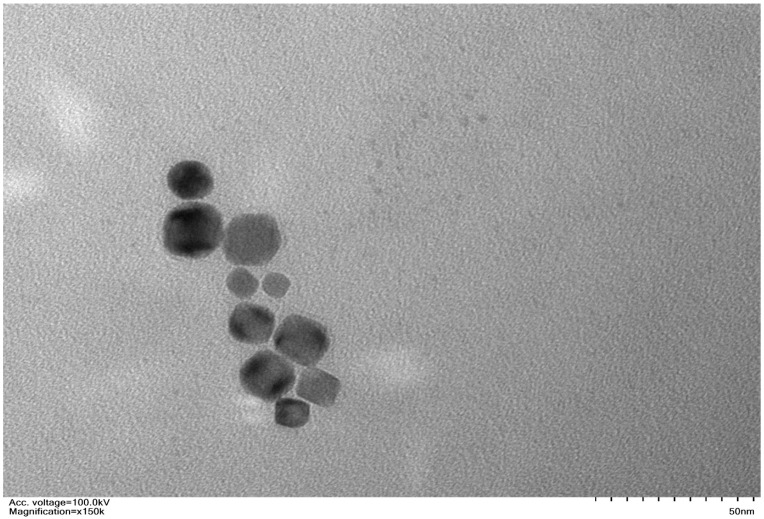
The transmission electron micrograph of the selenium particles showing spherical to quasi spherical shapes. For imaging purpose, a FEI Tecnai 12 apparatus fitted with a digital camera from Gatan was used. The particles were dried on a copper grid coated with a carbon layer.

Dark orange to dark red SeNPs were supplied by Sigma Aldrich (Product No. 919519). According to the manufacturer, inductively coupled plasma–mass spectrometry (ICP–MS) was carried out to comprehend the elemental composition, which confirmed the presence of Se. According to the suppliers, the size distribution of SeNPs ranges between 70 and 90 nm. In order to better understand the geometry of SeNPs, the Department of Botany, University of Gujrat, Pakistan conducted a transmission electron microscopic (TEM) examination. The results revealed that the SeNPs particles were spherical to quasi-spherical in shape (Figure 7). Various SeNPs concentrations, including trials at 25, 50, 75, and 100 ppm, as well as 0 ppm as the control treatment, were created for the seed priming treatment following [22]. To guarantee homogeneous dispersion, SeNPs were exposed to ultrasonication for 30 min. In the control treatment, the seeds were submerged in distilled water. All priming procedures were carried out for roughly 24 h in total at 25 °C.

The primed seeds were germinated in five separate seed trays corresponding to each priming treatment regime (i.e., tray one with 0 ppm treated seeds, trays 2, 3, 4, and 5 planted with seeds primed with 25, 50, 75, and 100 ppm concentrations of SeNPs. The seed trays were then placed in a growth chamber with fluorescent lighting (140 mmol m^−2^·S^−1^), a photoperiod of 16/8 h (light/dark period), a temperature of 24/18 °C (day/night), and regular irrigation with tap water every two days. At the three-leaf stage, tomato plants were transferred to the field for the drought experiment [77].

Two major plots (5 m × 3 m) were created in the experiment’s allotted space, one for each irrigation scheme (i.e., water and water stress). Five (1 m × 3 m) subplots were then created for each main plot, containing plants, one for each distinct seed priming treatment (i.e., 0, 25, 50, 75, and 100 ppm). Three equal-sized rows made up the subplots, which are referred to as replicates. So, three plants (one plant from each row) were selected for different analysis. There was no guardrail installed. Three rows of tomato plants were raised in each subplot of each major plot with a 45-cm intra- and 90-cm inter-row spacing, respectively [22].

In accordance with Dewis and Freitas [78], the soil’s physical and chemical properties were investigated. The soil used in the experiment had a clay loam texture, a 28.3% field capacity, and a 14% permanent wilting point. The soil type was vertosol. The soil’s pH was 7.6, and its electrical conductivity was 1.48 dSm^−1^ (1:5 H_2_O). It had 7.7 mg of organic carbon per gramme, 4.1 mg of extractable NO_3_ -N, 1.1 mg of extractable NH_4_-N, and 116 mg kg^−1^ of total P.

Drought stress was imposed to the water stressed plot following deficit irrigation principle proposed by Capra et al., [79]. Throughout the duration of the trial (twelve weeks), the tomato plants received full water applications (2 inches of water a week) as part of the water treatment. Treatment for water stress includes giving the plants full water for the first week, then withholding water for two weeks, carrying the same routine till the experiment lasted [79].

### 4.2. Observations on Growth Parameters

The data on growth attributes of experimental tomato plants were collected at crop maturity. The plant height and stem diameter were recorded using a measuring tape. Observations on shoot and root fresh weights of tomato plants were also recorded [22].

### 4.3. Determination of MDA Contents

A total of 1 g of freshly obtained leaf material was crushed in 10 mL of tricarboxylic acid (TCA) (10% solution in distilled water (dH_2_O)). About 2 mL of 0.5% thiobarbituric acid (TBA), produced in 20% TCA, was added to the supernatant (0.5 mL) that was recovered from the homogenized material. Test tubes containing the triturate were heated to 95 °C for 50 min before being immediately submerged in cold water to cool them. The absorbance of the colored portion was measured at 600 and 532 nm following centrifugation of the mixture (10,000× *g*) for 10 min [80].

MDA (nmol) = ∆ (A 532 nm − A 600 nm)/1.56 × 105

The absorption coefficient for the calculation of MDA is 156 mmol^−1^cm^−1^.

### 4.4. Determination of Hydrogen Peroxide Contents

In 10 mL of TCA (6%), the fresh tomato leaf sample (0.5 g) was thoroughly crushed. About 1 mL of a 1 M KI solution was added to 0.1 mL of centrifuged TCA extract. At 390 nm, the absorbance of the final combination was measured [81].

### 4.5. Activities of Antioxidative Defence Enzymes

SOD activity was assessed using the nitro blue tetrazolium (NBT) method under the photochemical reduction inhibition principle. About 560 nm was used to test NBT’s decreased inhibition. About 50 mM NBT, 75 nM ethylenediamine tetraacetic acid (EDTA), 50 mL enzyme extract, and 50 mM phosphate buffer were the ingredients in the study’s produced mixture, which contained 1.3 mM riboflavin, 13 mM methionine, and other ingredients (pH 7.8). The mixture was positioned under a fluorescent light (20 W) for 15 min in a box that has been coated with aluminum foil. Afterwards, a UV-visible spectrophotometer set to 560 nm was used to measure the reaction mixture’s absorbance [82].

As a fundamental phenomenon underlying the assessment of CAT activity, the mixture’s decreased absorbance was interpreted as the elimination of H_2_O_2_. The reaction mixture contained 100 L of extract, 1.9 mL H_2_O_2_, and 1 mL dH_2_O [83].

### 4.6. Determination of Leaf Chlorophyll Contents

Utilizing 80% acetone, the pigments were extracted as following [84]. A spectrophotometer was used to measure the extract’s absorbance at 663, 645, and 480 nm after fresh leaf material (0.1 g) was chopped and placed in 10 mL of 80% acetone for an overnight incubation at 4 °C (Hitachi U-2001, Tokyo, Japan). Amounts of carotenoids were analyzed using the formula described by the authors in [85].

### 4.7. Determination of Ascorbic Acid (AsA) Contents

In 10 mL of a 6% trichloroacetic acid solution, fresh leaf (0.25 g) was thoroughly pulverized. The supernatant was then treated with a 2 mL solution of 2% acidic dinitrophenyl hydrazine after being centrifuged at 10,000× *g* for 10 min. The triturate was next mixed with one drop of 10% thiourea. In a water bath, the resulting mixture was boiled for 20 min. After cooling to 0 °C, the liquid received 5 mL of an addition of 80% H_2_SO_4_. The finished colored material’s absorbance (OD) was measured at 530 nm. A standard curve was created from a range of standard solutions (50–300 ppm) by estimating the concentration in the leaf sample samples using pure AsA [86].

### 4.8. Determination of Total Anthocyanin Contents

Fresh leaf material (50 mg) was thoroughly homogenized in 1000 L of acidic MeOH (1% HCl, *w*/*v*) in an ice bath for the measurement of leaf anthocyanin concentration. At ambient temperature, the homogenate was centrifuged for five minutes at 14,000 rpm. At 657 and 530 nm, the supernatant’s OD was measured [87]. The following equation was used to calculate the amount of total anthocyanins content:

Total anthocyanins = (A530 − 0.25 × A657) × M^−1^

where A657 and A530 are the absorption at specific wavelengths, and M is the fresh leaf mass used for the extraction (g).

### 4.9. Total Flavonoid Contents

About 500 mg of freshly cut leaf material were crushed and extracted in ethyl alcohol at 25 °C. The content of flavonoids was estimated using the colorimetric technique. Catechin extract served as the benchmark for the calibration curve. Using a spectrophotometer, the absorbance was measured at 510 nm [88].

### 4.10. Determination of Proline Contents

After being homogenized with sulfosalicylic acid for 500 mg, the fresh leaf sample was centrifuged at 12,000× *g* for 8 min. A 2 mL aliquot of the supernatant was added to equal parts acid ninhydrin and glacial acetic acid at 100 °C for 60 min before the pellet was discarded. Toluene blue was employed to remove the proline from the slurry’s samples before 520 nm absorbance measurements were taken. A standard curve was used to calculate the proline contents [89].

### 4.11. Analysis of Glutathione S-Transferase (GST) Functioning

The approach of Hasanuzzaman and Fujita [90] was used to estimate GST activity. After combining 100 mM Tris-HCl, buffer (pH 7.0), 1 mM GSH, 1 mM 1-chloro-2,4-dinitrobenzene (CDNB), and enzyme extract, the reaction mixture was ready. A spectrophotometer was used to measure the absorbance at 340 nm (Beckman 640 D, Brea, CA, USA).

### 4.12. Determination of Ascorbate-Glutathione Pathway Enzymes Activity

According to Nakano and Asada [91], APX activity was measured. Ascorbate, H_2_O_2_, EDTA, and enzyme extract were added to a solution mixture that had a pH of 7.0. A spectrophotometer was used to measure the H_2_O_2_-mediated oxidation of ascorbate for two minutes at 290 nm (Beckman 640-D, USA).

The Foster and Hess method [92] was used to measure the activity of GR. The reaction mixture included enzyme extract, 100 mM potassium phosphate buffer (pH 7.0), 1 mM EDTA, 500 mM GSSG, and 150 mM NADPH. A spectrophotometer measured the change in absorbance color intensity for three minutes at 340 nm (Beckman 640-D, USA). The method of [93] was used to determine the MDHAR activity. MDHAR activity was measured in EU mg^−1^ protein (mol NADPH oxidized) units. Nakano and Asada [91] method was used to assess the DHAR activity in accordance with the established protocol. A spectrophotometer was used to measure the absorbance at 265 nanometers (Beckman 640-D, USA).

### 4.13. Determination of Tocopherols

A modified version of the Bakers’ [94] approach was used to measure the content of leaf tocopherols. Each sample’s fresh leaf material (0.5 g) was homogenized in 10 mL of a 2:1 (*v*/*v*) petroleum ether and ethanol solution before being centrifuged at 10,000× *g* for 20 min. One milliliter of an aliquot was combined with 200 L of 2-dipyridyl in ethanol (2%) and carefully mixed before being kept in the dark for five minutes. After properly mixing, 4 mL of distilled and deionized water were added to the mixture. A spectrophotometer set at 520 nm was used to get the reading. The tocopherol content was calculated using a standard curve built with known concentrations of tocopherol.

### 4.14. Measurement of Reduced and Oxidised Glutathione Contents

Following [95], the amounts of reduced and oxidized glutathione in leaves were measured. Briefly, 250 mg of fresh leaf material were thoroughly milled in 2 mL of 0.1 M HCl containing 1 mM EDTA. The extract was then centrifuged at 12,000× *g* for 15 min at 4 °C to get the supernatant. The reaction mixture was made by combining 200 mL of phosphate buffer (125 mM), 100 mL of DTNB (6.0 mM), 200 mL of extract, and 500 mL of NADPH (0.3 mM). At 412 nm, the mixture’s absorbance was measured.

### 4.15. Statistical Analysis

All the experiments were conducted in completely randomized block design. Utilizing Co-STAT, data were subjected to an analysis of variance (ver. 6.3, Cohort Software, Berkeley, CA, USA). XLSTAT (ver. 2014.5.03, Addinsoft 1995–2014, Paris, France) was employed to create the Pearson correlation matrix and principal component analysis.

## 5. Conclusions

In the present research, we tested our hypothesis that SeNPs-mediated tomato seed priming can strengthen antioxidant defense and confer drought tolerance. The tomato plants grown from SeNPs-primed seeds in the field trials demonstrated superior growth and biomass accumulation in terms of shoot and root fresh weights, plant height, and stem diameters. The water-stressed plants raised from primed seeds had decreased H_2_O_2_ and MDA contents. Moreover, it was shown that the antioxidant enzymes SOD and CAT had increased activity. In tomato plants developed from SeNPs-primed seeds, it was discovered that non-enzymatic antioxidants such as tocopherols, glutathione, carotenoids, flavonoids, and anthocyanins were boosted. The tomato plants were able to effectively combat the threat of drought due to SeNPs-mediated seed priming, which boosted antioxidant defense enzymes such as APX, GST, GR, MDHAR, and DHAR and preserved redox status in tomato plants. In fragile areas, seed priming might be a practical method to boost crop productivity. To explore the potential function of nano priming, the authors suggest conducting more research.

## Figures and Tables

**Table 1:** Physiochemical parameters of tomato plants measured after seed priming using SeNPs and analyzed through ANOVA (with mean square and *p* values).

Variation Source	^a^ *df*	SFW	RFW	PH	TC	Carotenoids	Ascorbic Acid	α-Tocopherols
Water Stress (WS)	1	4788.033 ^b^*** (0.000)	375.948 *** (0.000)	533.408 *** (0.000)	2.616 *** (0.000)	13.912 *** (0.000)	4966.345 *** (0.000)	421.12 *** (0.000)
Se-NPs Seed Priming (SSP)	4	421.033 *** (0.000)	37.052 *** (0.000)	64.122 *** (0.000)	0.151 *** (0.000)	0.449 *** (0.000)	602.8 *** (0.000)	22.418 *** (0.000)
WS X SSP	4	10.033 ns (0.120)	0.692 ns (0.464)	0.464 ns (0.537)	0.004 * (0.021)	0.006 ns (0.263)	63.553 *** (0.000)	0.722 ns (0.162)
Error	20	4.800	0.742	0.577	0.001	0.004	5.566	0.394
Variation Source	*df*	Flavonoids	H_2_O_2_	MDA	SOD	CAT	GST	APX
Water Stress (WS)	1	95.551 *** (0.000)	12,120.3 *** (0.000)	811.2 *** (0.000)	8433.633 *** (0.000)	5266.871 *** (0.000)	1954.392 *** (0.000)	574.918 *** (0.000)
Se-NPs Seed Priming (SSP)	4	8.425 *** (0.000)	622.133 *** (0.000)	64.466 *** (0.000)	816.783 *** (0.000)	186.366 *** (0.000)	57.153 *** (0.000)	28.238 ns (0.057)
WS X SSP	4	0.717 ** (0.013)	201.8 ***(0.000)	22.533 *** (0.000)	183.716 *** (0.000)	36.416 *** (0.000)	2.099 ns (0.079)	8.535 ns (0.522)
Error	20	0.105	7.666	2.633	9.366	2.991	0.855	0.001
Variation Source	*df*	GR	MDHAR	DHAR	GSH	GSSG	Proline	Anthocyanins
Water Stress (WS)	1	2043.030 *** (0.000)	1560.984 *** (0.000)	5826.920 *** (0.000)	5096.33 *** (0.000)	594.965 *** (0.000)	459.425 *** (0.000)	0.633 *** (0.000)
Se-NPs Seed Priming (SSP)	4	48.010 *** (0.000)	11.872 *** (0.000)	169.671 *** (0.000)	492.283 ***(0.000)	42.492 ***(0.000)	20.122 *** (0.000)	0.019 *** (0.000)
WS X SSP	4	8.617 *** (0.000)	2.417 ns (0.339)	20.401 *** (0.000)	67.783 *** (0.000)	6.362 ** (0.001)	5.468 *** (0.000)	0.006 *** (0.000)
Error	20	0.763	2.033	2.528	6.133	0.929	0.272	0.001

^a^ *df*: degree of freedom, ns: non-significant, H_2_O_2_: hydrogen peroxide; RFW: root fresh weights; SFW: shoot fresh weights; PH: plant height; TC: total chlorophyll; MDHAR: monodehydroascorbate reductase; DHAR: dehydroascorbate reductase; GST: glutathione s transferase; APX: ascorbate peroxidase; DR: glutathione reductase; CAT: catalase; MDA: malondialdehyde; H_2_O_2_: hydrogen peroxide; SOD: superoxide dismutase; GSH: reduced glutathione; GSSG: oxidized glutathione. ^b^ *, **, and *** = significant at 0.05, 0.01, and 0.001 levels, respectively.

**Table 2:** Spearman correlation matrix showing significant correlation among various physiochemical parameters of tomato plants raised through SeNPs-primed seeds.

Variables	TC	Carotenoids	Ascorbic Acid	Toco	Flavonoids	H_2_O_2_	MDA	SOD	CAT	GST	APX	GR	MDHAR	DHAR	GSH	GSSG	Proline	Ant
TC	1																	
Carotenoids	−0.517 *	1																
Ascorbic acid	−0.512 *	0.989 *	1															
Toco	−0.52 *	0.989 *	0.993 *	1														
Flavonoids	−0.517 *	0.995 *	0.991 *	0.985 *	1													
H_2_O_2_	−0.983 *	0.532 *	0.532 *	0.537 *	0.531 *	1												
MDA	−0.936 *	0.591 *	0.573 *	0.577 *	0.59 *	0.924 *	1											
SOD	−0.492 *	0.966 *	0.972 *	0.966 *	0.973 *	0.511 *	0.539 *	1										
CAT	−0.52 *	0.97 *	0.98 *	0.97 *	0.978 *	0.54 *	0.57 *	0.972 *	1									
GST	−0.518 *	0.989 *	0.995 *	0.991 *	0.988 *	0.537 *	0.581 *	0.97 *	0.976 *	1								
APX	−0.522 *	0.812 *	0.794 *	0.798 *	0.799 *	0.508 *	0.595 *	0.788 *	0.769 *	0.793 *	1							
GR	−0.528 *	0.973 *	0.972 *	0.955 *	0.982 *	0.547 *	0.575 *	0.973 *	0.978 *	0.967 *	0.774 *	1						
MDHAR	−0.513 *	0.995 *	0.99 *	0.988 *	0.989 *	0.524 *	0.581 *	0.964 *	0.969 *	0.989 *	0.799 *	0.965 *	1					
DHAR	−0.521 *	0.989 *	0.977 *	0.974 *	0.988 *	0.528 *	0.589 *	0.972 *	0.957 *	0.979 *	0.808 *	0.972 *	0.986 *	1				
GSH	−0.519 *	0.989 *	0.982 *	0.977 *	0.988 *	0.525 *	0.589 *	0.962 *	0.968 *	0.985 *	0.795 *	0.967 *	0.99 *	0.986 *	1			
GSSG	−0.51 *	0.967 *	0.985 *	0.983 *	0.969 *	0.534 *	0.571 *	0.951 *	0.957 *	0.976 *	0.782 *	0.942 *	0.969 *	0.952 *	0.955 *	1		
Proline	−0.513 *	0.985 *	0.992 *	0.981 *	0.99 *	0.531 *	0.573 *	0.982 *	0.976 *	0.987 *	0.799 *	0.979 *	0.986 *	0.987 *	0.98 *	0.973 *	1	
Ant	−0.521 *	0.969 *	0.978 *	0.965 *	0.977 *	0.525 *	0.574 *	0.961 *	0.971 *	0.971 *	0.839 *	0.969 *	0.975 *	0.97 *	0.974 *	0.954 *	0.981 *	1

H_2_O_2:_ hydrogen peroxide; TC: total chlorophyll; MDHAR: monodehydroascorbate reductase; DHAR: dehydroascorbate reductase; GST: glutathione s transferase; APX: ascorbate peroxidase; DR: glutathione reductase; CAT: catalase; MDA: malondialdehyde; H_2_O_2_: hydrogen peroxide; SOD: superoxide dismutase; GSH: reduced glutathione; GSSG: oxidized glutathione; Toco: α-tocopherols; Ant: anthocyanin. * Significantly different from 0 at a significance level of alpha = 0.05.

## Data Availability

All data are available within this publication.
